# Water Stability of Fibers-Enhanced Asphalt Mixtures under Static and Dynamic Damage Conditions

**DOI:** 10.3390/ma17061304

**Published:** 2024-03-12

**Authors:** Yue Xiao, Tianlei Wang, Zongwu Chen, Chao Li, Feng Wang

**Affiliations:** 1 State Key Laboratory of Silicate Materials for Architectures, Wuhan University of Technology, Wuhan 430070, China; xiaoy@chd.edu.cn (Y.X.); wangtianlei@whut.edu.cn (T.W.); lchao@whut.edu.cn (C.L.); 2 School of Materials Science and Engineering, Chang’an University, Xi’an 710061, China; wangf2312@chd.edu.cn; 3 Faculty of Engineering, China University of Geosciences (Wuhan), Wuhan 430074, China; 4 Key Laboratory for Special Area Highway Engineering of Ministry of Education, Chang’an University, Xi’an 710064, China

**Keywords:** asphalt mixture, dynamic water pressure, fiber, void rate

## Abstract

Water damage is one of the major distresses of asphalt pavements. Existing methods for investigating the water stability of asphalt mixtures rely primarily on static water test methods, the tensile strength ratio (TSR) test, and the retained Marshall stability (RMS) test, which evaluate the strength and stability loss after freeze-thaw damage or hot water immersion, respectively. However, these methods do not accurately replicate the actual dynamic water damage conditions to pavement. Therefore, in this study, a variety of damage conditions, including static water conditions and dynamic water pressure conditions, were used to investigate the effects of lignin fibers (LFs), polyester fibers (PFs), and polypropylene fibers (PPFs) on the water stability of asphalt mixtures. First, three fibers-enhanced SMA gap-gradation asphalt mixtures were designed. Then, TSR and RMS were measured under traditional static water damage conditions and new dynamic water pressure damage conditions to evaluate the effect of fiber types on the water stability of asphalt mixtures. Finally, the void rate of asphalt mixtures and its changes under dynamic water damage conditions were further revealed with the help of CT scanning technique. Results showed that, among these three types of fibers, PFs-enhanced asphalt mixture exhibited excellent stability under both static and dynamic water conditions, and the CT scanning test also indicated that the PFs can significantly reduce the increase rate of voids in asphalt mixtures after dynamic water pressure damage. This study identified the potential of incorporating suitable type of fiber to enhance the performance of asphalt mixture under dynamic water pressure damage.

## 1. Introduction

Water is one of the main factors causing various types of distresses in the early service stage of asphalt pavement [1]. It is well accepted that there are two main forms of damage caused by water to asphalt pavement [2]; one is to weaken the adhesion between asphalt and aggregates, and the other is to deteriorate the internal cohesion of asphalt [3,4,5]. Water not only causes loss of cohesion, but also decreases the adhesion between asphalt and aggregate, and this decrease in adhesion affects many properties of asphalt pavements [6,7,8].

For asphalt pavement in service, the damage caused by water is actually a very complex process. Studies have shown that free water infiltrating into the interior of asphalt pavement structure will form super-porous water flow under the action of vehicle load, and the asphalt pavement undergoing the dual action of load and pore water scouring finally generates structural damage [9,10,11]. Therefore, the effect of dynamic water pressure cannot be ignored for asphalt pavement in service. With the continuous construction of asphalt pavement, especially the application of porous asphalt pavement, the water damage caused by dynamic water pressure to asphalt pavement is becoming more and more prominent [12]. The dynamic water pressure accelerates the adhesion loss and cohesion loss. This is because the liquid water in the pavement structure can flow freely in the channel connecting the voids, and the asphalt pavement will gradually dense under the repeated load of the vehicle, so that the connected voids lose permeability, which will produce excessive pore water pressure, and such void water pressure will accelerate the stripping of asphalt and aggregate, resulting in moisture damage [13].

Currently, the water stability of asphalt mixture is mainly evaluated under the static water damage conditions. In the static water evaluation methods, two types of test specimens are used; one is uncompacted asphalt mixture (asphalt or asphalt mastic coated aggregate, loose asphalt mixture, etc.), and the other is compacted asphalt mixture (Marshall specimen, cored specimen, etc.) [14,15]. The water stability of asphalt mixture is determined based on the loss of asphalt of uncompacted asphalt mixture and the loss of Marshall stability, indirect tensile strength, etc. Although these methods are simple to operate, the water-soaked or freeze-thaw conditions cannot well simulate the field site water damage conditions, especially the dynamic water pressure caused by the vehicle load. In practice, water damage of asphalt pavement is a gradually increasing process; the repeated action of the vehicle load will generate circulating dynamic water pressure inside the voids, and asphalt mixture in this process will be continuously scoured, resulting in the gradual accumulation of damage and then developing into water damage [16,17]. Therefore, it is necessary to consider the effect of dynamic water pressure when evaluating the water damage resistance of asphalt mixture. Considering the dynamic water pressure will damage the internal structure of asphalt mixture [18], analyzing water stability from the changes in structure of asphalt mixture such as voids would be highly efficient, Mohammed et al. [19] used a plastic-modified asphalt mixture, and in the experiment, it was found that the void rate decreased as the plastic content increased. The water stability of the mixture increases with the increase of plastic content, which indicates that the water resistance of the asphalt mixture may be related to the change of its void rate. In addition, Sanfilippo et al. [20] prepared asphalt mixtures with different voids and performed X-ray scans before and after freezing and thawing, and they found that the properties of the asphalt mixture were closely related to the void characteristics. CT technology can detect the internal structure of the object without contact; find the internal defects of the product quickly, accurately, and intuitively; and analyze them, so as to find the root cause of the defect. In fact, as early as 2007, Arambula et al. [21] used CT technology to analyze the internal void characteristics of asphalt mixture and found that the water stability of asphalt mixture is closely related to the spatial distribution of internal voids. On this basis, Alawneh et al. [22] collected nine ray slices at different parts of the asphalt mixture before and after the freeze-thaw cycle to replace the overall internal structure of the sample and used ImageJ software (https://imagej.net/ij/download.html, bundled with 64-bit Java 8) to analyze to evaluate the change law of the internal voids of the sample, Omranian et al. [23] converted the CT image into a 3D model and detected the structural failure of the asphalt mixture by the color threshold method. However, although CT technology is well established for analyzing the structural changes of asphalt mixtures after water damage, at present, there is still little CT image analysis for the large number of asphalt mixtures, especially the lack of CT scanning studies of asphalt mixtures based on dynamic water quality testing methods.

There are many ways to improve the performance of asphalt mixtures, which can be done by laying hands on the asphalt, through SBS modification and composite material modification, etc. [24,25,26]; it can also be directly improved in the mixture by adding additives, such as hydrated lime, rubber, fiber, etc. [27,28,29]. It has been shown that the introduction of fibers in asphalt mixtures can enhance the strength and stiffness of the mixtures [30], giving them higher modulus, viscosity, water stability, high-temperature stability, low-temperature crack resistance, and durability, which prolongs the service life of asphalt mixtures as pavement materials [31,32]. Qin et al. [33] examined the effect of basalt fibers of varied lengths (6 mm, 9 mm, and 15 mm) and contents (3–10%) on the properties of asphalt mastics and found out that the addition of basalt fibers generally improved the properties of asphalt mastics especially the crack resistance. Tang et al. [34] explored the effect of alkali-treated waste betel nut fiber (BNF) on the water stability of asphalt mixture; the results showed that for the AC-13 gradation, the optimal combination of multi-response variables was a fiber content of 0.2561%, fiber shear time of 12.78 min, and asphalt-aggregate rate of 5.82%. Abdelsalam et al. [35] carried out a laboratory study on the properties of asphalt mixture modified with a new composite composed of diatomaceous earth powder and lignin fibers. Results revealed that the use of the composite in asphalt mixture led to an enhancement in the asphalt pavement performance. Previous studies have confirmed that the reliability of using fibers to improve a series of properties of asphalt mixtures. It also indicates that using fibers to strengthen the water damage resistance of asphalt mixture has advantages because many other performances of asphalt mixture can also be synchronously enhanced. Among the many types of fibers, lignin fibers, polyester fibers, and polypropylene fibers are not only more common but also have better economic benefits.

Based on the above, in order to fully understand the water damage characteristics and improve the water stability of asphalt mixture, in this research, multiple water damage conditions were used. In addition to the traditional static water-soaked condition and water freeze-thaw condition, the dynamic water pressure condition was also adopted. The fiber was also used to enhance asphalt mixture. Firstly, fiber enhanced asphalt mixtures were designed by Marshall method, and three types of fibers, namely lignin fibers (LFs), polyester fibers (PFs), and polypropylene fibers (PPFs), were involved. Then, the moisture stability of fiber enhanced asphalt mixtures was further investigated. It included two tasks; for static water conditions, the RMS test (determining the loss of Marshall stability of asphalt mixture after hot water-soaked damage) and the TSR test (determining the loss of splitting tensile strength of asphalt mixture after freeze-thaw damage) were conducted. For dynamic water pressure conditions, besides the dynamic water RMS test and TSR test, X-ray CT scanning technology with image recognition and processing software ImageJ was further used to extract and analyze voids, and the influence of fibers on the water damage resistance behavior of asphalt mixture was analyzed based on the distribution feature of voids and the change rule of voids after the dynamic water scouring test.

In this paper, by combining the traditional static water conditions and dynamic water conditions test methods, we can not only achieve a comprehensive evaluation of asphalt mixture performance but also compare the differences between the two methods. In addition, by improving the previous test method, the CT scan image of asphalt mixture is divided into three different analysis parts, which can better describe its overall characteristics. With the help of dynamic water condition test, the improvement of water stability by adding fibers into asphalt mixtures can be also forwarded.

## 2. Materials and Methods

### 2.1. Materials

#### 2.1.1. Fibers

**Figure 1:**
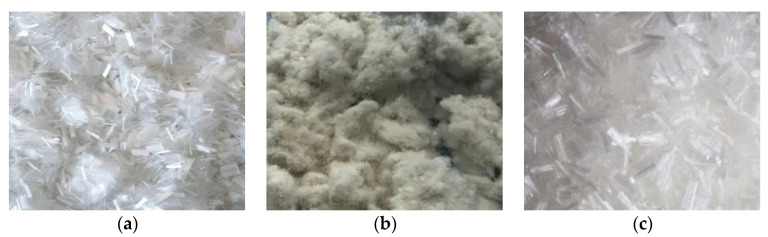
Used fibers ((**a**): PFs; (**b**): LFs; (**c**): PPFs).

Three fibers (PFs, LFs PPFs), produced by the Hubei Luxiang Chemical Technology Co., Ltd. (Jingzhou, China), were selected in this research, and the appearance of these three fibers is shown in Figure 1. It can be seen that PFs and PPFs were dispersed, while LFs were flocculent.

The technical properties of these three fibers, tested according to the standard test methods of the Chinese technical specification [36], are shown in Table 1, Table 2 and Table 3. In fact, there is no big gap between the international standards for fiber testing methods and the Chinese standards, and the results showed that all the technical properties of selected fibers met the requirements of the Chinese technical specification. The lignin fibers used in this paper had a maximum length of 4.7 mm and a minimum length of 2.6 mm, polypropylene fibers had a length of 12 mm and a diameter of 20 μm, and polyester fibers had a length of 20 mm and a diameter of 18 μm.

#### 2.1.2. Asphalt Binder

The asphalt binder used in this research was SK Speedway-modified asphalt from Korea. Its penetration, softening point, and ductility were also measured according to the Chinese standard test methods [37], and results are listed in Table 4. It showed that the tested technical properties of asphalt binder also met the requirements of the Chinese technical specification.

#### 2.1.3. Aggregate and Filler

The aggregate and filler used in this research were from Jingshan, Hubei, China. The coarse aggregate was basalt, fine aggregate was limestone, and filler was limestone powder. The technical properties of aggregate and filler were also carried out according to the Chinese standard test methods [38]. The test results were presented in Table 5, Table 6 and Table 7, which also indicated that all the technical properties of basalt coarse aggregate, limestone fine aggregate, and limestone powder filler met the requirements of the Chinese technical specification.

#### 2.1.4. Hybrid Gradation of Mineral Raw Materials

**Figure 2:**
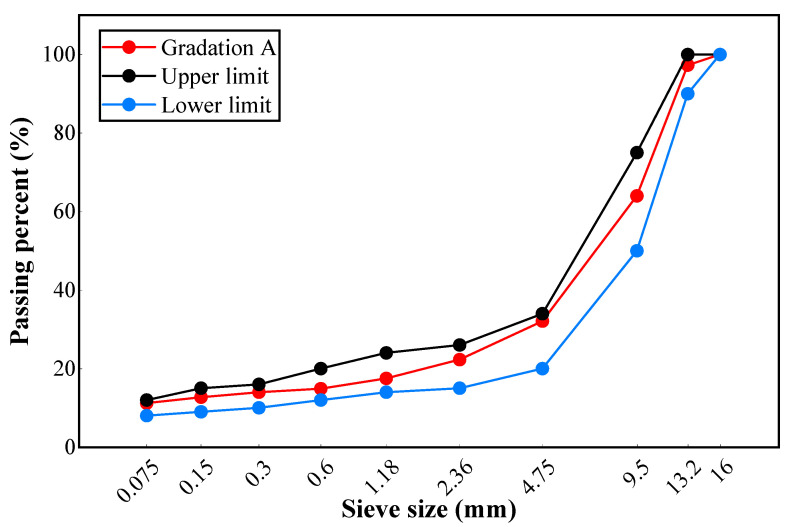
The hybrid gradation curve.

A gap-graded stone mastic asphalt (SMA) mixture with a maximum nominal particle size of 13.2 mm was designed according to Marshall design method. The hybrid gradation of mineral raw materials is shown in Table 8 and Figure 2.

The mineral materials were first mixed according to gradation A, and then the Marshall test was conducted after mixing the mineral mixture with three different asphalt-aggregate rates. According to the SMA pavement design specification [37], the empirical asphalt-aggregate rate was set to the median, and then 0.3% was added and subtracted from it. These asphalt-aggregate rates were used as experimental values. Results are shown in Table 9. It can be seen that the air void rate of asphalt mixture under the asphalt-aggregate rate of 6.1% cannot meet the design requirements [39]. Although all the design indicators of asphalt mixtures under the other two asphalt-aggregate rate of 6.4% and 6.7% were satisfactory, considering the cost, 6.4% was preferred as the optimum asphalt-aggregate rate. After determining the asphalt-aggregate rate, three types of fibers were added to create fiber-enhanced asphalt mixtures, the fiber content was determined to be 0.3% of the total asphalt mixture. The fiber-enhanced asphalt mixture molding method in this study was as follows: First, we dried the fibers at 100 °C with constant mass, and then mixed the aggregates according to the design gradation and dried them at 165 °C for 4 h. The dried aggregates and fibers were added to the asphalt mixing pot for 90 s to ensure a homogeneous mixing of the aggregates and fibers, and finally, the heated asphalt was added to the dynamically inflowed mixing pot and stirred again for 90 s, to obtain the formulated fibrous asphalt mixture. Finally, the fiber-enhanced asphalt mixture was obtained.

### 2.2. Methods

#### 2.2.1. Research Program

**Figure 3:**
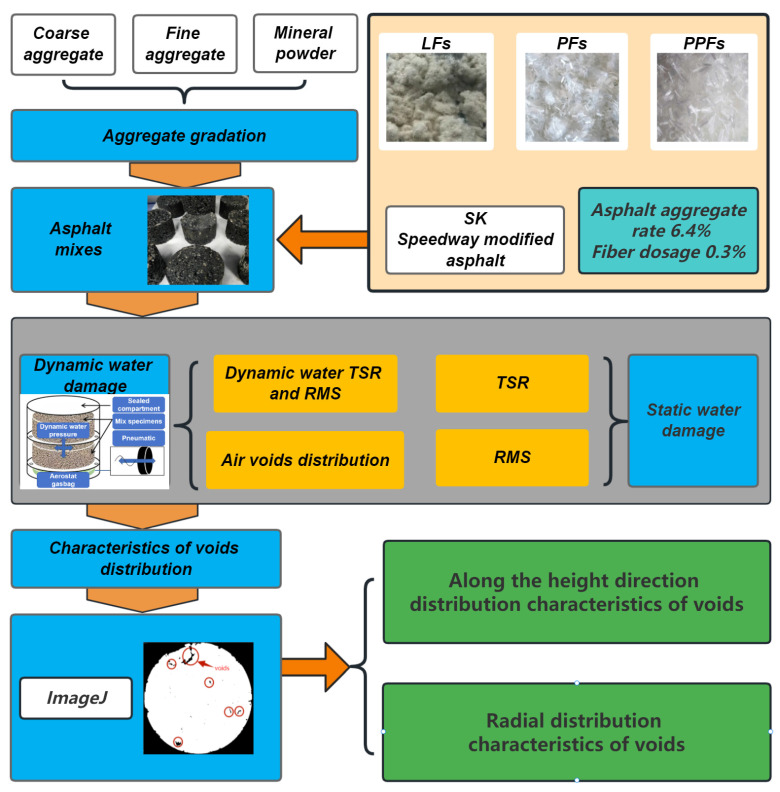
Study plan of this research.

The technical route of this study is shown in Figure 3. Firstly, SMA mixtures containing PFs, PPFs, and LFs, respectively, were designed by the Marshall method. Then, the water stability of asphalt mixtures was investigated by static water methods and new methods considering dynamic water pressure, respectively. Static water methods included the RMS test and TSR test, and dynamic water pressure methods included the dynamic water RMS test, TSR test, and a CT scanning technique based on the void analysis test incorporating image recognition and processing software of ImageJ. ImageJ was used to analyze the distribution feature of voids and the change rule of voids after the dynamic water scouring test, so as to further evaluate the effect of fiber on the water damage resistance of SMA mixtures.

#### 2.2.2. Static Water Test Methods

##### TSR Test

The tensile strength ratio (TSR) value can show the freeze–thaw damage resistance ability of the asphalt mixture. The TSR test was conducted according to the Chinese standard method [38]. Eight Marshall specimens with a diameter of 101.6 mm and a height of 63.5 mm for each asphalt mixture were prepared, and they were randomly divided into two groups on average. They functioned as the control group and conditioned group, respectively. All specimens in the conditioned group were first subjected to vacuum saturation operation, which was kept under vacuum of 98 kPa for 15 min, and then they were sent to a freezer for freezing at −18 °C for 16 h, and finally, all specimens were moved to a hot water bath of 60 °C and soaked for 24 h. After that, both groups of specimens were put into a water bath of room temperature about 25 °C for 2 h, and then all Marshall specimens were sent to splitting test with the UTM-130 multifunctional testing system. The test was carried out at 25 °C, and the loading speed was 50 mm/min. The splitting tensile strength was determined according to Equation (1) as follows:

(1) $$R_{T}=\frac{0{.006287P}_{T}\;}{h}$$

where *R_T_*—splitting tensile strength (MPa); *P_T_*—peak load (N); *h*—the height of the specimen (mm).

The freeze–thaw split tensile strength ratio was calculated according to Equation (2) as follows:

(2) $$TSR=\frac{{\bar{R}}_{T2}}{{\bar{R}}_{T1}}\times 100$$

where *TSR*—tensile strength ratio (%); *R_T_*_1_—average splitting tensile strength of control group (MPa); *R_T_*_2_—average splitting tensile strength of the conditioned group (MPa).

##### RMS Test

The retained Marshall stability (RMS) value can show the hot water damage resistance ability of asphalt mixture. Specimens of the same size as the TSR test were prepared and used for each asphalt mixture; eight Marshall specimens were also randomly divided into two groups on average. The specimens in the control group were sent to a hot water bath of 60 °C and soaked for 30–40 min, and the specimens in the conditioned group were soaked in the same hot water bath of 60 °C for 48 h. Both groups of specimens were also sent to measure Marshall stability with the UTM-130 multifunctional testing system. The RMS was computed by Equation (3):

(3) $$RMS=\frac{MS_{2}}{MS_{1}}\times 100$$

where *RMS*—retained Marshall stability (%); *MS*_1_—average Marshall stability of the control group (kN); *MS*_2_—average Marshall stability of the conditioned group (kN).

#### 2.2.3. Test Methods Considering Dynamic Water Pressure

##### Dynamic Water TSR and RMS Test

**Figure 4:**
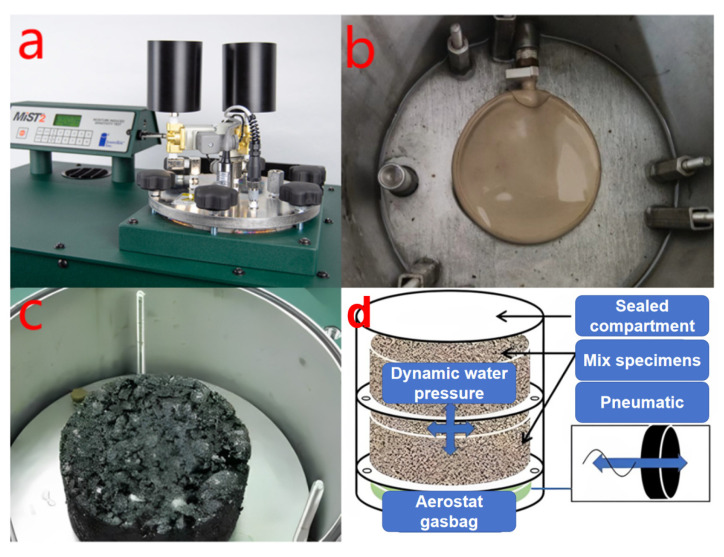
MIST instrument and schematic diagram of its principle ((**a**): MIST instrument; (**b**): airbag at the bottom of the instrument; (**c**): Marshall specimen loaded on the upper partition board; (**d**): working principle).

As stated in the introduction section, dynamic water has an important effect on the performance of asphalt pavements. In order to apply dynamic water pressure to the Marshall specimens, a MIST from Instroke in Boston, MA, USA was used. The MIST is designed to simulate the damage behavior of repeated action of water and traffic loads to asphalt pavement. As shown in Figure 4a, the left side was the operation panel of MIST, and the right side was the top of the sealing chamber; the internal sealing chamber of MIST consisted of an airbag at the bottom and two partition boards. Figure 4b shows the airbag at the bottom, Figure 4c shows the Marshall specimen loaded on the upper partition board, and Figure 4d shows the working principle of MIST, which is that MIST controls the water at different temperatures through the sealing chamber to generate the pore pressure and simulate the movement of automobile tires on wet pavement.

In dynamic water pressure test, same as the static water test method, Marshall specimens were used. For each asphalt mixture, a total of 24 specimens were prepared, half for the dynamic water TSR test and half for the dynamic water RMS test. For each test, 12 specimens were randomly divided into three groups on average. One group functioned as control group, and the other two groups functioned as conditioned groups. The two conditioned groups of specimens were rinsed 3500 cycles at a dynamic water pressure of 30 Psi and 50 Psi, respectively. The temperature of water was 60 °C. After that, all specimens in control group and conditioned groups were sent to the splitting test or Marshall stability test. The dynamic water TSR and RMS can be calculated by Equations (4) and (5), respectively, and it can be seen that the calculation method was similar to that in the static water method.

(4) $${TSR}_{i}=\frac{R_{Ti}}{R_{T}}\times 100$$

where *TSR_i_*—dynamic water tensile strength ratio of the asphalt mixture after damage with a dynamic water pressure of *i* Psi (%); *R_T_*—average splitting tensile strength of the control group (MPa); *R_Ti_*—average splitting tensile strength of the conditioned group after damage with a dynamic water pressure of *i* Psi (MPa).

(5) $$RMS_{i}=\frac{MS_{i}}{MS}\times 100$$

where *RMS_i_*—dynamic water retained Marshall stability of the asphalt mixture after damage with a dynamic water pressure of *i* Psi (%); *MS*—average Marshall stability of the control group (kN); *MS_i_*—average Marshall stability of the conditioned group after damage with a dynamic water pressure of *i* Psi (kN).

##### CT Scanning Analysis

**Figure 5:**
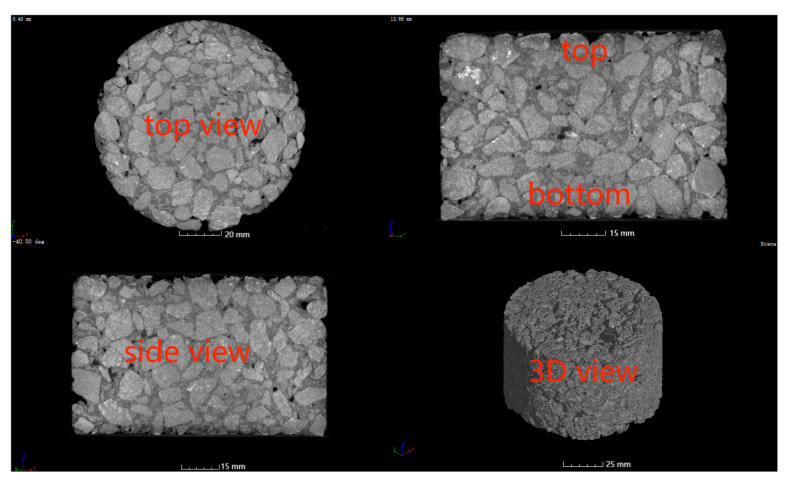
Schematic of CT scanning direction.

In this study, a GE Vtomex industrial CT scanning equipment with a scanning voltage of 180 kV, a current of 200 μA, and a resolution of 54 μm was used to analyze the void distribution feature of Marshall specimens and their change after the dynamic water pressure scouring damage was applied. The CT scanning direction is shown in Figure 5.

**Figure 6:**
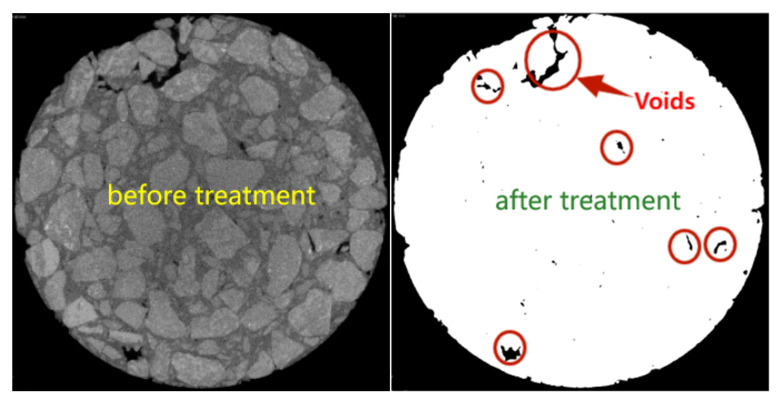
Image before and after processing by ImageJ.

ImageJ software was applied to identify and analyze the void portion of the cross-sectional image along the height and radius direction of specimen, so as to obtain the distribution pattern of voids inside the specimen. In ImageJ software, the void part of the image was extracted after a series of image processing processes to select a suitable threshold value. The ct images were binarized, and each substance had a specific grayscale value, ranging from 0 to 255. The gray value of the gap was 0. In this study, the emphasis was not on the analysis of aggregate and adhesive asphalt, but only on the analysis of the voidage of asphalt mixture, so there was no excessive adjustment to the image, and the distribution of voidage could be clearly obtained in the gray threshold of about 10–70. The images before and after processing are shown in Figure 6.

**Figure 7:**
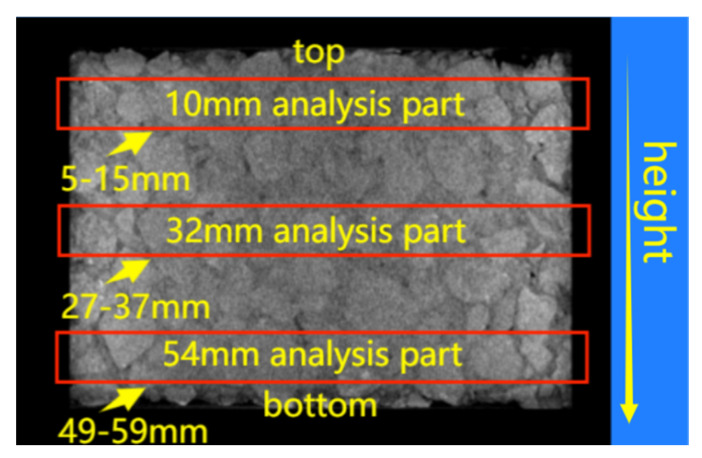
Selection of analytical parts.

The void rate of each section can be determined by dividing the void area by the cross-sectional area. So, it was easy to obtain void characteristics along the height direction of specimen based on the total void of each cross-section. When analyzing the voids along the radius direction of specimen, three cross-sectional images at 10 mm, 32 mm, and 54 mm from top to bottom along the height direction of the specimen were selected. In order to overcome the non-representative problem of void distribution of a single cross-sectional image, the images were extended 5 mm upward and downward at each selected cross-sectional position, and finally, an analysis part with a thickness of 10 mm was used for each cross section, as shown in Figure 7.

## 3. Results and Discussion

### 3.1. Static Water Test Results

#### 3.1.1. TSR Results

**Figure 8:**
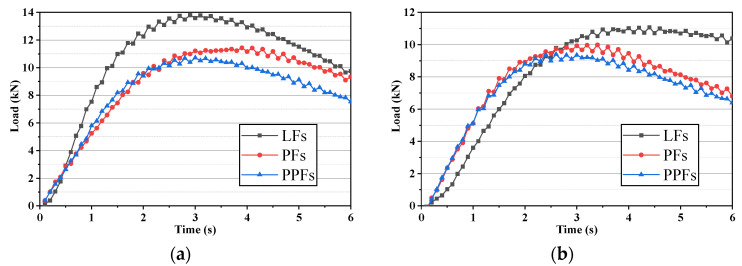
Load bearing capacity curves of fibers-enhanced asphalt mixtures ((**a**): before freeze-thaw damage; (**b**): after freeze-thaw damage).

In splitting test, the load bearing capacity curves of different fibers-enhanced asphalt mixtures are shown in Figure 8, which showed that the peak loads of all three fibers-enhanced asphalt mixtures decreased to different degrees after the freeze-thaw damage, while the size relationships between the three peak loads did not change. Among them, LFs-enhanced asphalt mixture had a largest peak load before and after the freeze-thaw damage, while the peak load of PPFs-enhanced asphalt mixture was the smallest, and PFs-enhanced asphalt mixture had a peak load between their peak loads.

**Figure 9:**
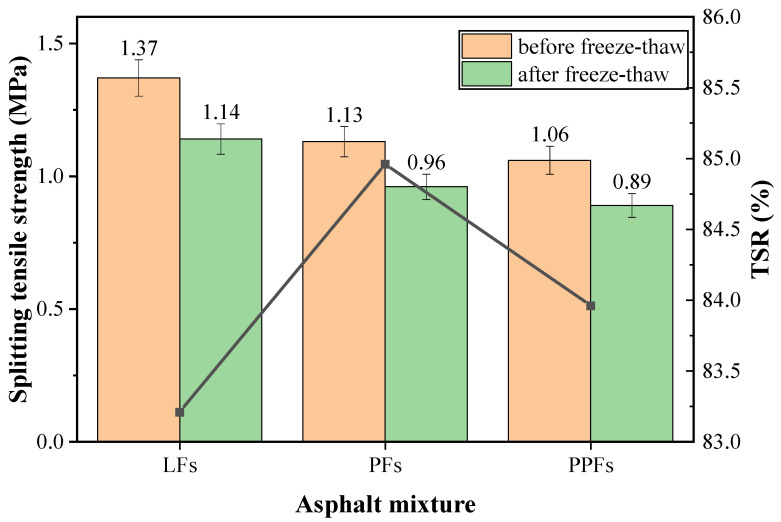
Splitting tensile strength and TSR before and after freeze–thaw damage.

The splitting tensile strength of three different fiber enhanced asphalt mixtures were calculated based on the peak loads. The splitting tensile strength and TSR results of asphalt mixtures containing different fibers are shown in Figure 9. It can be found that the splitting tensile strengths of three different fibers-enhanced asphalt mixtures were various. The original asphalt mixture containing LFs possessed the uppermost splitting tensile strength of 1.37 MPa, which was 21.2–29.2% higher than that of the other two fiber enhanced asphalt mixtures. It indicated that LFs played a positive role in improving the splitting tensile strength of asphalt mixture. This was because the LFs was finer than PPFs and PFs (see Figure 1), and a larger number of LFs were more closely interconnected in the asphalt mixture, which will be very helpful for improving the bonding performance within asphalt mixture system. When the splitting failure was applied, LFs can provide more resistance, which made the splitting tensile strength of LFs-enhanced asphalt mixture even bigger. For PFs and PPFs, the splitting tensile strengths of PPFs-enhanced asphalt mixture were less than that of PFs-enhanced asphalt mixture, this may be because the melting point of PPFs was lower than that of PFs, which was only 165 °C, when mixed and prepared asphalt mixtures, PPFs may partially melt due to high mixing temperature, resulted in fewer fibers for enhancement.

The splitting tensile strengths of three asphalt mixtures obviously decreased after the freeze–thaw damage. In detail, the PFs-enhanced asphalt mixture showed the highest TSR of 85.0%, while the LFs-enhanced asphalt mixture showed the lowest TSR of 83.2%, although LFs can improve the splitting tensile strength of original asphalt mixture obviously. The TSR of asphalt mixture reflects its stability against freeze–thaw damage: the higher the TSR is, the stronger the freeze–thaw damage resistance is. Therefore, PFs did better than LFs and PPFs in improving the freeze–thaw damage resistance of the asphalt mixture.

#### 3.1.2. RMS Results

The stability and RMS results of the three fibers-enhanced asphalt mixtures are shown in Table 10. It can be found that the asphalt mixture with PFs always had the highest stability value before and after hot water-soaked damage, and they were 17.55 kN and 17.09 kN, respectively. It indicated that the RMS of the PFs-enhanced asphalt mixture was upped to 97.38%. Although the Marshall stabilities of the LFs- and PPFs-enhanced asphalt mixtures before or after hot water immersion were lower than that of the PFs-enhanced asphalt mixture, the RMS values of the former two asphalt mixtures were still higher than 90%. In detail, the RMS of the LFs-enhanced asphalt mixture and PFs-enhanced asphalt mixture was relatively close and significantly higher than the RMS of the PPFs-enhanced asphalt mixture, which was only 91.9%. Therefore, PFs and LFs did better in improving the hot water damage resistance of asphalt mixture.

### 3.2. Test Results under Dynamic Water Pressure

#### 3.2.1. Dynamic Water TSR and RMS

**Figure 10:**
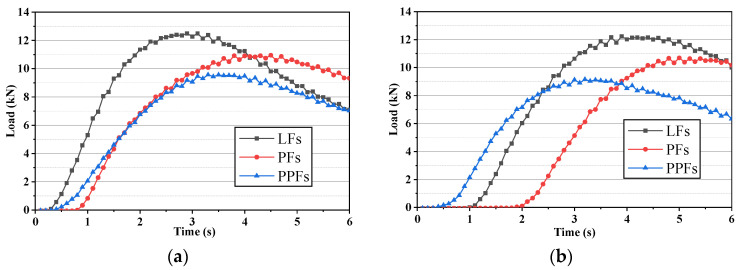
Load-bearing capacity curves of the fibers-enhanced asphalt mixtures after dynamic water pressure damage ((**a**): after 30 Psi dynamic water pressure damage; (**b**): after 50 Psi dynamic water pressure damage).

In splitting tests, the load-bearing capacity curves of these three asphalt mixtures mixed with different fibers before and after dynamic water rinsing are shown in Figure 10, For each fiber-enhanced asphalt mixture, it can be found that the peak load was not much different after 30 Psi and 50 Psi dynamic water pressure damage. However, there were still significant differences in the peak load of different asphalt concretes. Similar to the results in the static water TSR test, the LFs-enhanced asphalt mixture possessed the biggest peak load whether after 30 Psi or 50 Psi dynamic water pressure damage, which was higher than 12 kN. The peak load of the PPFs-enhanced asphalt mixture was the smallest, which was generally below 10 kN. The PFs-enhanced asphalt mixture also had a peak load between their peak loads.

**Figure 11:**
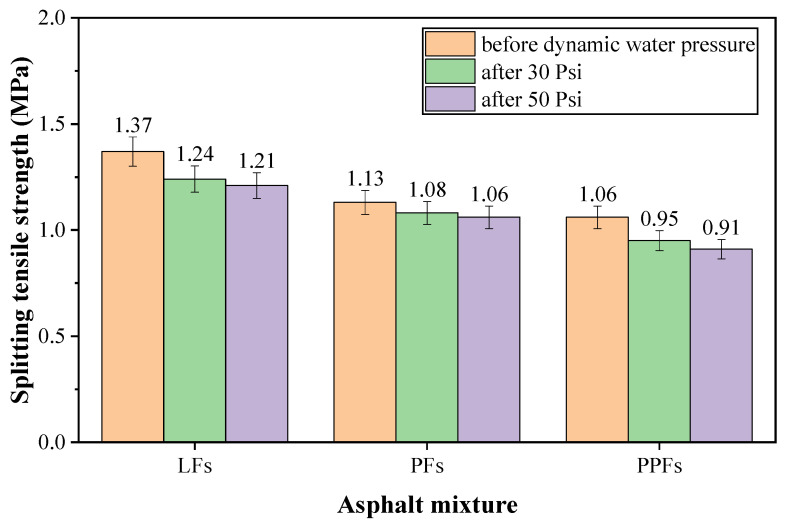
Splitting tensile strength of the fibers-enhanced asphalt mixture before and after dynamic water pressure.

The dynamic water splitting tensile strength results are shown in Figure 11. It can be found that, under the same experimental condition, the LFs-enhanced asphalt mixture always possessed the highest splitting tensile strength, followed by the strength of the PFs-enhanced asphalt mixture, and the strength of the PPFs-enhanced asphalt mixture was the lowest. The strength loss caused by dynamic water pressure damage is shown in Table 11. It can be seen that although the splitting tensile strength of the asphalt mixtures showed a decreasing trend with the increase of dynamic water pressure, there were significant differences in the strength variation characteristics of the three asphalt mixtures. In detail, compared to the damage condition of dynamic water pressure of 30 Psi, the TSR values of asphalt mixtures after damage with dynamic water pressure of 50 Psi were lower to varying degrees, which indicated that the destructive effect of dynamic water pressure on asphalt mixtures was tremendous. The decrease of the strength of the PPFs-enhanced asphalt mixture was the biggest among these three asphalt mixtures, whose TSR was just 85.85% after damage with dynamic water pressure of 50 Psi. It agreed with load results shown in Figure 10. The PFs-enhanced asphalt mixture showed the best dynamic water damage resistance. Its TSR can still reach up to 93.81% even after damage with dynamic water pressure of 50 Psi. Dynamic water TSR results also showed that the ability of the LFs-enhanced asphalt mixture to resist the dynamic water pressure damage was between that of the PFs-enhanced asphalt mixture and PPFs-enhanced asphalt mixture. Therefore, in terms of the dynamic water TSR results, the asphalt mixture incorporated with PFs possessed better durability under dynamic water pressure damage than the other two fibers-enhanced asphalt mixtures.

The Marshall stability and RMS of the asphalt mixtures after dynamic water pressure damage are shown in Table 12, Clearly, the Marshall stability of each fiber-enhanced asphalt mixture also decreased after dynamic water pressure damage, and it suggested that Marshall stability of asphalt mixture was sensitive to dynamic water pressure damage. While the PPFs-enhanced asphalt mixture was also more sensitive to the change of dynamic water pressure, when the dynamic water pressure increased from 30 Psi to 50 Psi, its RMS decreased from 87.34% to 83.63% with a decrease rate of 4.25%. Compared to the PPFs-enhanced asphalt mixture, the other two fibers-enhanced asphalt mixtures showed higher dynamic water damage stability, and even after damage with dynamic water pressure of 50 Psi, the RMS values of the LFs-enhanced asphalt mixture and PFs-enhanced asphalt mixture were both higher than 95%. PFs did a little better than LFs in improving the dynamic water damage resistance of asphalt mixture. So, according to the dynamic water RMS results, PFs also had the highest potential in enhancing the resistance of asphalt mixture to dynamic water pressure damage among these three fibers.

**Figure 12:**
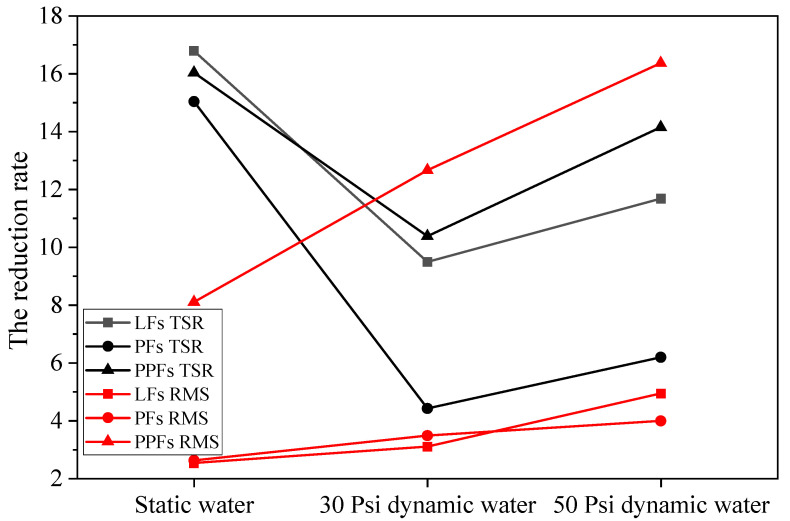
Comparison of the test results under dynamic and static water conditions.

In addition, the reduction rates of TSR and RMS for the three fibers under different test conditions are shown in Figure 12, and it can be found that compared with the results of the static water test, the three fiber asphalt mixtures obtained under the dynamic water test method had lower reduction rates of TSR, while the reduction rates of RMS were on the high side. Undoubtedly, there was a difference in the results obtained under the two test methods, and it is undoubtedly closer to the actual situation under the dynamic water method, so it is necessary to promote the dynamic water test method.

#### 3.2.2. Air Voids Distribution Characteristics

##### Distribution Characteristics of Voids along the Height Direction

Based on the above experiment results, especially in Figure 12, it can be found that the degradation rate of various properties of the PFs-reinforced asphalt mixture was relatively low; it can be concluded that the overall performance of the PFs-enhanced asphalt mixtures was relatively better than the other two fibers-enhanced asphalt mixture. It may be more instructive to observe its internal structure, so this mixture was selected for CT scanning test to analyze the effect of fiber on the dynamic water erosion resistance of asphalt mixture. Under the dynamic water pressure of 50 Psi, the TSR and RMS of the asphalt mixture specimens changed significantly, so the PFs-enhanced asphalt mixture under the experimental condition of dynamic water pressure with 50 Psi was used in the follow-up test.

**Figure 13:**
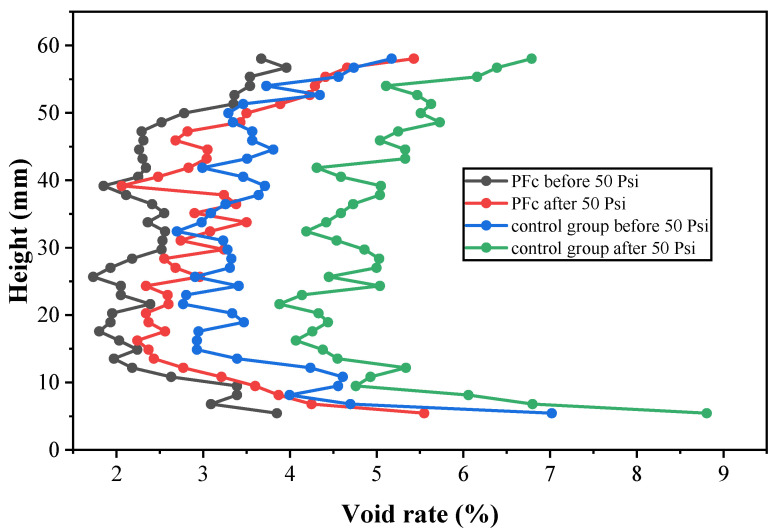
Distribution of voids in height direction before and after dynamic water pressure damage.

The asphalt mixture without fiber was used as control group, and the asphalt mixture containing PFs was used as the conditioned group. The two groups of specimens were cyclically rinsed 3500 times at 60 °C with dynamic water pressure of 50 Psi. The distribution of the internal voids along the height direction of the Marshall specimen before and after dynamic water pressure damage is shown in Figure 13. As can be seen from Figure 13, the internal voids of the specimen were unevenly distributed along the height direction. In detail, the voids were roughly symmetrically distributed except for the upper and lower ends of the specimen, and the void rates of the upper and lower ends of the specimen were significantly higher than that of the central region. This was related to the movement ability of aggregate particles during the compaction process of asphalt mixture. When preparing the Marshall specimen, the loose asphalt mixture was first placed into a cylindrical mold, and then a Marshall hammer struck the upper surface of asphalt mixture. During this process, the aggregate particles in contact with the bottom surface of the mold and the aggregate particles of upper surface in contact with the hammer had difficulty changing their position in space and could only adjust their posture on the original two-dimensional plane. Therefore, the flatness at both ends of the specimen was poor. This difference led to an edge effect, where the edges were less compacted.

As can be seen from Table 13, after the addition of PFs, the void rate of the specimen decreased, indicating that the addition of PFs had a positive effect on reducing the internal void rate of the asphalt mixture, and the increase of the void rate after the addition of PFs was smaller than that of the control group, indicating that PFs could effectively improve the resistance of the asphalt mixture to dynamic water erosion.

##### Radial Distribution Characteristics of Voids

**Figure 14:**
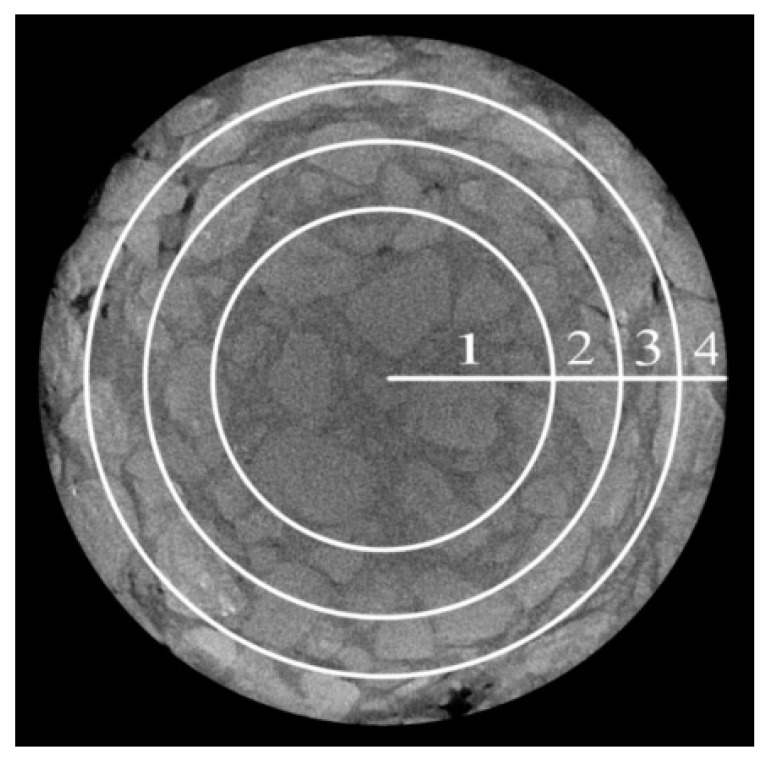
Radial segmentation zones.

The radial distribution characteristics of the voids was analyzed based on the area equivalents as shown in the Figure 14. The cross-sectional image was segmented into four parts by three circles with a radius of $\frac{R}{2}$, $\frac{\sqrt{2}R}{2}$, $\frac{\sqrt{3}R}{2}$, respectively, and *R* was the radius of specimen. The four segmented zones were numbered from inside out as 1, 2, 3, and 4, respectively.

**Figure 15:**
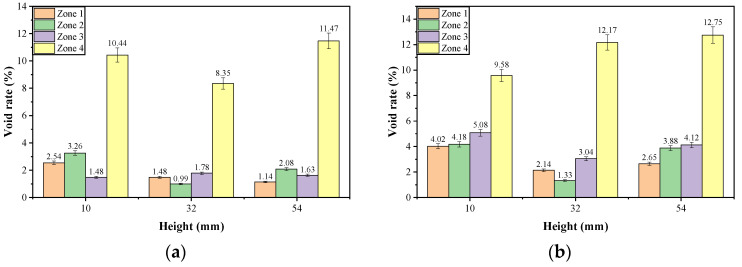

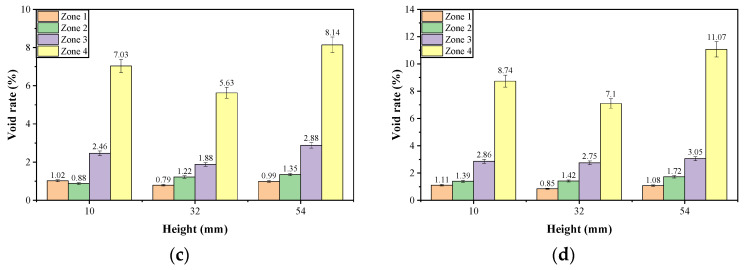
Radial distribution of voids of asphalt mixture ((**a**): control group before 50 Psi; (**b**): control group after 50 Psi; (**c**): PFs-conditioned group before 50 Psi; (**d**): PFs-conditioned group after 50 Psi).

For these four segmented zones of each part with a thickness of 10 mm as shown in Figure 7 and Figure 14, the average void rate of every zone was determined using ImageJ to analyze several scanning cross-sectional images within the height range of 10 mm. The statistical results of the void rates of asphalt mixtures before and after dynamic water pressure damage are shown in Figure 15 and Table 14. On the whole, Table 14 showed that, whether before or after dynamic water pressure damage, the total void rates of the three parts of the PFs-enhanced asphalt mixture specimen were all lower than that of the control group specimen. Figure 15 displays the specific distribution of void in different zones of each part; for the PFs-enhanced asphalt mixture specimen, the void rate roughly showed an increasing trend from zone 1 to zone 4 for each part. In contrast, the distribution of voids in the control group was more complex, and there was no obvious rule. Although the void rate from zone 1 to zone 4 did not show a significant change pattern, the void rate of zone 4 was obviously higher than that of the other three zones, which may also be related to the edge effect. After the dynamic water pressure test, except for zone 4 in the 10 mm cross-sectional analysis part (the height range of 5–15 mm), for the control group, all void rates of other zones were increased to varying degrees. For a certain part, dynamic water pressure damage may cause some zones to expand while others may be compressed; that is why there was a decrease in void rate of the zone just mentioned. At the same time, for these zones affected by expansion, the expansion effects in these zones were inconsistent, which also resulted in varying degrees of void rate changes in these zones after dynamic water damage.

Table 14 also shows that the void growth rates of all three analysis parts of the PFs-enhanced asphalt mixture were lower than those of the control asphalt mixture. In detail, the void growth rates of the three analysis parts of the PFs-enhanced asphalt mixture ranged from 23.59% to 26.65%, and for the control asphalt mixture, the corresponding void growth rate ranged from 28.67% to 48.25%. It also suggests that the control group had larger fluctuations in the growth rate of voids after damage with a dynamic water pressure of 50 Psi. Therefore, the total void rate results of the original asphalt mixtures and void growth rate results of asphalt mixtures after dynamic water pressure damage indicate that PFs can not only reduce the void rate of asphalt mixture but also significantly improve the water damage resistance of asphalt mixtures.

## 4. Conclusions

In this study, three types of fibers, LFs, PFs, and PPFs, were used to prepare fiber-enhanced SMA asphalt mixtures. The TSR and RMS of these three fibers-enhanced asphalt mixtures were first measured under static water damage conditions and dynamic water pressure conditions, and then the CT scanning test was further used to analyze the void characteristics of asphalt mixture under dynamic water pressure condition. The main conclusions of the study were as follows:

(1) Results of traditional static water TSR and RMS tests suggested that PFs endowed the asphalt mixture with excellent water stability. For the PFs-enhanced asphalt mixture, its TSR value was the highest, reaching 85%. Although its RMS value was a little lower than that of the LFs-enhanced asphalt mixture, the difference was quite small; they were 97.38% and 97.47%, respectively.

(2) Results of the dynamic water TSR and RMS tests showed that the splitting tensile strength and Marshall stability of asphalt mixtures decreased with the increase of dynamic water pressure, and the PFs-enhanced asphalt mixtures possessed larger TSR and MRS even after damage with high dynamic water pressure of 50 Psi, which were 93.81% and 96.01%, respectively.

(3) The distribution characteristics of voids in the height and radius directions analyzed by the CT scanning technique both indicated that the void rate of the PFs-enhanced asphalt mixture was smaller than that of the fiber-free control group, and after applying dynamic water pressure, the increase in the total void rate of the PFs-enhanced asphalt mixture was 25.49% lower than the control group.

The dynamic water simulation method adopted in this study is closer to the actual condition of asphalt mixture, and the test results obtained based on this method are more accurate, which is conducive to guiding the formulation of subsequent research and the development of asphalt pavement.

## Figures and Tables

**Table 1:** Technical properties of lignin fibers (LFs).

Parameters	Technical Requirements	Test Results
0.15 mm quality pass rate (%)	60–80	72
Ash content (%)	13–23	18
pH value	6.5–8.5	7.1
Oil absorption rate (multiplier times)	5–9	7
Water content (%)	≤5	2.4
Mass loss (%, 210 °C, 1 h)	≤6, and no combustion	3.1
Wood fiber content (%)	≥85	94.8
Maximum length (mm)	≤6	4.7
Average length (mm)	-	2.6
Density (g/cm^3^)	-	1.10

**Table 2:** Technical properties of polypropylene fibers (PPFs).

Parameters		Technical Requirements	Test Results
Lengths	Average value (mm)	10–38	12
	Misalignment (%)	±10	-
Calibre	Average value (μm)	15–35	20
	Misalignment (%)	±10	-
Rupture strength (MPa)		≥800	980
Elongation at break (%)		≥8.0	15
Density (g/cm^3^)		0.910 ± 0.040	0.91
Curly fiber content (%)		≤3	1.2
Melting point (°C)		≥160	165

**Table 3:** Technical properties of polyester fibers (PFs).

Parameters		Technical Requirements	Test Results
Lengths	Average value (mm)	19–38	20
	Misalignment (%)	±10	-
Calibre	Average value (μm)	10–20	18
	Misalignment (%)	±10	-
Rupture strength (MPa)		≥450	508
Elongation at break (%)		≥20	25
Density (g/cm^3^)		1.360 ± 0.050	1.38
Curly fiber content (%)		≤3	1.2
Melting point (°C)		≥240	255

**Table 4:** Technical properties of the asphalt binder.

Parameters	Technical Requirements	Test Results
Penetration@25 °C, 100 g, 5 s (0.1 mm)	50–60	58.6
Ductility@5 cm/min, 5 °C (cm)	≥20	23.8
Softening point (°C)	≥60	79.1

**Table 5:** Technical properties of the basalt coarse aggregate.

Parameters	Technical Requirements	Test Results	
		4.75–9.5 mm	9.5–16 mm
Apparent relative density	≥2.6	2.961	2.953
Water absorption (%)	≤2.0	0.8	0.6
Needle flake content (%)	≤10	1.8	1.5
Crush value (%)	≤24	10.6	
Los Angeles abrasion (%)	≤28	16.8	
Polishing value	≥42	53	
Adhesion level with asphalt	≥5	5	

**Table 6:** Technical properties of the limestone fine aggregate.

Parameters	Technical Requirements	Test Results
Apparent relative density	≥2.5	2.650
Sand equivalent (%)	≥60	64
<0.075 mm particle content (%)	≤1	0.5
Robustness (%)	≥12	14
Angular (flow time, s)	≥30	42

**Table 7:** Technical properties of the limestone powder filler.

Parameters		Technical Requirements	Test Results
Apparent density (g/m^3^)		≥2.5	2.684
Passing percent of different particle size range (%)	<0.6 mm	100	100
	<0.15 mm	90–100	94.8
	<0.075 mm	75–100	83.8
Hydrophilicity		<0.9	0.68
Appearance		Solidarity-free block	Solidarity-free block

**Table 8:** The hybrid gradation of mineral raw materials.

Sieve Size (mm)		16.0	13.2	9.5	4.75	2.36	1.18	0.6	0.3	0.15	0.075
Passing percent (%)	Upper limit	100.0	100.0	75.0	34.0	26.0	24.0	20.0	16.0	15.0	12.0
	Lower limit	100.0	90.0	50.0	20.0	15.0	14.0	12.0	10.0	9.0	8.0
	Gradation A	100.0	97.3	64.0	32.1	22.3	17.5	14.9	14.0	12.7	11.2

**Table 9:** Volumetric properties of the gradation A asphalt mixture volumetric properties.

Gradation	Asphalt-Aggregate Rate (%)	Bulk Relative Density	Maximum Theoretical Relative Density	Air Voids (%)	VMA (%)	VFA (%)	VCA_mix_ (%)
A	6.1	2.475	2.583	4.2	18.1	76.9	40.7
	6.4	2.479	2.572	3.6	18.2	80.1	40.7
	6.7	2.474	2.561	3.4	18.6	81.7	40.8
Requirements	-	-	-	2–4	≥17.0	75–85	≤VCA_DRC_

Note: VMA—voids in mineral aggregate; VFA—voids filled with asphalt; VCA_mix_—coarse aggregate skeleton clearance rate; VCA_DRC_—void rate of coarse aggregate in compacted condition, test result 43.5%.

**Table 10:** Stability and RMS results of the fiber-enhanced asphalt mixtures.

Fiber Type	Marshall Stability of Control Group (kN)	Marshall Stability of Conditioned Group (kN)	RMS (%)
LFs	15.81	15.41	97.47
PFs	17.55	17.09	97.38
PPFs	16.43	15.10	91.90

**Table 11:** Results of the dynamic water TSR.

Fiber Type	TSR_30_ (%)	TSR_50_ (%)
LFs	90.51	88.32
PFs	95.58	93.81
PPFs	89.62	85.85

**Table 12:** Results of the dynamic water Marshall stability and RMS.

Type	After 30 Psi (kN)	RMS_30_ (%)	After 50 Psi (kN)	RMS_50_ (%)
LFs	15.32	96.90	15.03	95.06
PFs	16.94	96.52	16.85	96.01
PPFs	14.35	87.34	13.74	83.63

**Table 13:** Total void rate of each group before and after dynamic water pressure damage.

Type	Void Rate (%)		Void Growth Percent (%)
	Before 50 Psi	After 50 Psi	
Control group	3.64	5.11	40.38
PFs conditioned group	2.55	3.20	25.49

**Table 14:** Void rate of different analysis parts before and after dynamic water pressure damage.

Parameters	Analysis Parts	Void Rate (%)		Void Growth Rate (%)
		Before 50 Psi	After 50 Psi	
Control group	10 mm	4.43	5.70	28.67
	32 mm	3.15	4.67	48.25
	54 mm	4.08	5.85	43.38
PFs conditioned group	10 mm	2.84	3.51	23.59
	32 mm	2.38	3.01	26.47
	54 mm	3.34	4.23	26.65

## Data Availability

Data are contained within the article.
